# Successful removal of a proximally migrated pancreatic stent using a novel device delivery system

**DOI:** 10.1055/a-2063-3408

**Published:** 2023-04-17

**Authors:** Akihiro Matsumi, Kazuyuki Matsumoto, Daisuke Uchida, Shigeru Horiguchi, Koichiro Tsutsumi, Hironari Kato, Motoyuki Otsuka

**Affiliations:** Okayama University Graduate School of Medicine, Dentistry, and Pharmaceutical Sciences, Okayama, Japan

The removal of a proximally migrated pancreatic stent is technically challenging 1 2 3 . A novel device delivery system (EndoSheather; Piolax) was recently developed, which comprises a slim-tipped guide catheter and pusher tube that facilitate the insertion of devices up to 1.9 mm in diameter 4 5 .

An 80-year-old man was admitted to our hospital for treatment of choledocholithiasis. We removed the bile duct stone by performing endoscopic sphincterotomy; however, black stools were observed 2 days after the procedure. Emergency endoscopy revealed an exposed blood vessel in the papilla of Vater. We planned to perform hemostasis by injecting hypertonic saline and epinephrine after stenting both the bile and pancreatic ducts; however, the pancreatic stent (Geenen, 5 Fr, 3 cm; Cook Medical Japan) migrated during the procedure. We were unable to remove the migrated pancreatic stent despite attempts with several different devices, including a dilation balloon, stone removal balloon, and basket catheter, with the stent finally ending up in the tail of the pancreatic duct (PD) ( Fig. 1 a ). We therefore replaced the additional pancreatic stent, and the procedure was terminated once hemostasis had been achieved.

**Fig. 1:**
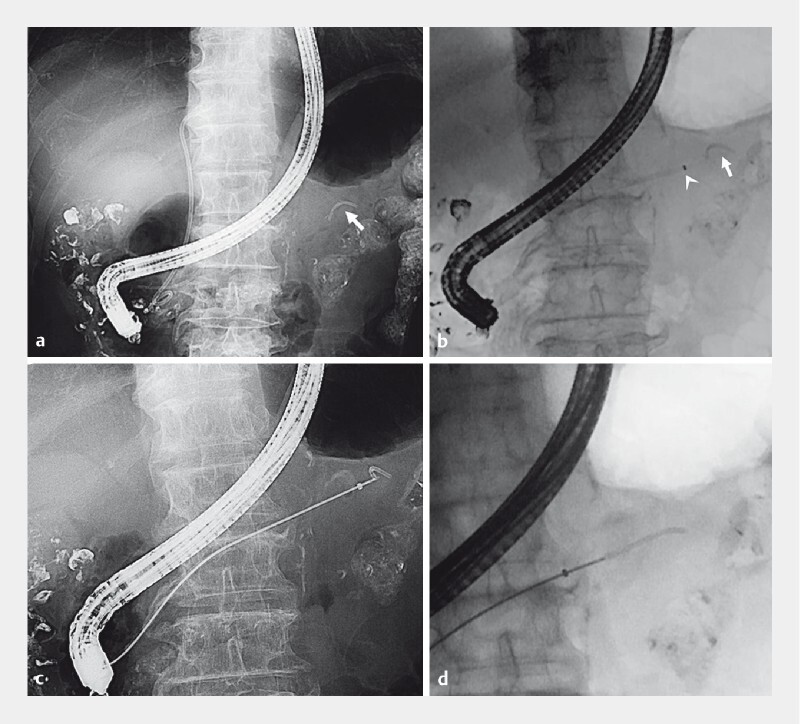
Fluoroscopic images showing: **a** the pancreatic stent (arrow) that had migrated into the tail of the pancreatic duct; **b** a novel device delivery system (EndoSheather; arrowhead) that was inserted close to the stent (arrow), with the inner catheter then removed; **c** the intraductal cholangioscopy forceps with a 1-mm diameter (SpyBite) being used to grasp the migrated stent; **d** the biopsy forceps being pulled up within the outer sheath.

Cessation of the bleeding was confirmed 2 days later, when we also attempted to remove the migrated pancreatic stent. First, a guidewire (EndoSelector; Boston Scientific) was placed on the proximal side of the PD. Second, the novel device delivery system (EndoSheather) was inserted close to the migrated pancreatic stent, and the inner catheter was removed ( Fig. 1 b ). A biopsy forceps with a 1.8-mm diameter (Radial Jaw; Boston Scientific) was then inserted through the sheath; however, it was not possible to open the jaws because of the narrow PD. We therefore used a smaller biopsy forceps with a 1-mm diameter (SpyBite; Boston Scientific) to grasp the migrated pancreatic stent ( Fig. 1 c ). Finally, we pulled the biopsy forceps up into the outer sheath of the device ( Fig. 1 d ) and were able to successfully remove the migrated stent ( Fig. 2 ). Endoscopic nasopancreatic drainage was subsequently performed to prevent pancreatitis due to clots ( Video 1 ), and the patient was discharged without further complications.

**Fig. 2:**
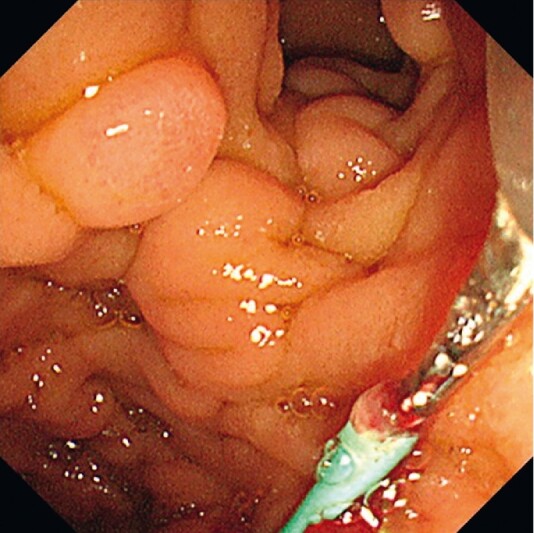
Endoscopic view showing the migrated stent being successfully grasped with the biopsy forceps and removed.

**Video 1** Successful removal of a proximally migrated pancreatic stent using a novel device system.


Endoscopy_UCTN_Code_TTT_1AR_2AZ

## Notes

Endoscopy E-Videos

https://eref.thieme.de/e-videos
*E-Videos* is an open access online section of the journal *Endoscopy* , reporting on interesting cases and new techniques in gastroenterological endoscopy. All papers include a high-quality video and are published with a Creative Commons CC-BY license. Endoscopy E-Videos qualify for HINARI discounts and waivers and eligibility is automatically checked during the submission process. We grant 100% waivers to articles whose corresponding authors are based in Group A countries and 50% waivers to those who are based in Group B countries as classified by Research4Life (see: https://www.research4life.org/access/eligibility/ ). This section has its own submission website at https://mc.manuscriptcentral.com/e-videos

## References

[1] Matsumoto K Katanuma A Maguchi H Endoscopic removal technique of migrated pancreatic plastic stents J Hepatobiliary Pancreat Sci 2014 21 E34 E40 24535753 10.1002/jhbp.94

[2] Ghimire S Ravi S J Yousef M Proximal migration of pancreatic duct stent in pancreas divisum: challenges in retrieval and review of the literature Case Rep Gastrointest Med 2021 2021 5.531658E6 10.1155/2021/5531658 PMC8081641 33968451

[3] Ng W K Tan Q R Punamiya S J Novel method to remove deeply migrated pancreatic duct stent Endoscopy 2022 54 E748 E749 35359016 10.1055/a-1792-2469

[4] Okada H Uza N Matsumori T A novel technique for mapping biopsy of bile duct cancer Endoscopy 2021 53 647 651 32961577 10.1055/a-1248-2138

[5] Matsumori T Uza N Shiokawa M Mapping biopsy for bile duct cancer using a novel device delivery system Endoscopy 2022 54 E217 E219 34058756 10.1055/a-1479-1969

